# Survey of Canadian Animal-Based Researchers' Views on the Three Rs: Replacement, Reduction and Refinement

**DOI:** 10.1371/journal.pone.0022478

**Published:** 2011-08-17

**Authors:** Nicole Fenwick, Peter Danielson, Gilly Griffin

**Affiliations:** 1 Canadian Council on Animal Care, Ottawa, Ontario, Canada; 2 W. Maurice Young Centre for Applied Ethics, University of British Columbia, Vancouver, British Columbia, Canada; Université Pierre et Marie Curie, France

## Abstract

The ‘Three Rs’ tenet (replacement, reduction, refinement) is a widely accepted cornerstone of Canadian and international policies on animal-based science. The Canadian Council on Animal Care (CCAC) initiated this web-based survey to obtain greater understanding of ‘principal investigators’ and ‘other researchers’ (i.e. graduate students, post-doctoral researchers etc.) views on the Three Rs, and to identify obstacles and opportunities for continued implementation of the Three Rs in Canada. Responses from 414 participants indicate that researchers currently do not view the goal of replacement as achievable. Researchers prefer to use enough animals to ensure quality data is obtained rather than using the minimum and potentially waste those animals if a problem occurs during the study. Many feel that they already reduce animal numbers as much as possible and have concerns that further reduction may compromise research. Most participants were ambivalent about re-use, but expressed concern that the practice could compromise experimental outcomes. In considering refinement, many researchers feel there are situations where animals should not receive pain relieving drugs because it may compromise scientific outcomes, although there was strong support for the Three Rs strategy of conducting animal welfare-related pilot studies, which were viewed as useful for both animal welfare and experimental design. Participants were not opposed to being offered “assistance” to implement the Three Rs, so long as the input is provided in a collegial manner, and from individuals who are perceived as experts. It may be useful for animal use policymakers to consider what steps are needed to make replacement a more feasible goal. In addition, initiatives that offer researchers greater practical and logistical support with Three Rs implementation may be useful. Encouragement and financial support for Three Rs initiatives may result in valuable contributions to Three Rs knowledge and improve welfare for animals used in science.

## Introduction

In Canada, the Canadian Council on Animal Care (CCAC) is the national organization with the responsibility for overseeing the care and use of animals in science. CCAC is mandated to act in the interests of the people of Canada 1) to ensure that the use of animals in science employs optimal care according to acceptable scientific standards and 2) to promote an increased level of knowledge, awareness and sensitivity to relevant ethical principles.

Studies show that the CCAC mandate is well-supported by the Canadian public. For example, Gauthier and Griffin [1] estimated that public acceptance of the use of animals in science is approximately 85% when regulation is in place and pain and distress are minimized. An opinion poll found that 66% of Canadians support human use of animals (including research use) “… as long as unnecessary pain and suffering are minimized” [2]. A study to determine the effect of regulation on public acceptance of animal-based science found that support for invasive procedures was higher when use was regulated versus not regulated [3]. As these examples illustrate, regulatory oversight is necessary to maintain the support of a public that both accepts the use of animals in science, and is concerned about animal welfare and minimizing pain and distress.

Minimizing pain and distress for animals used in science is also a key goal for Canadian granting agencies and Canadian researchers. This is reflected by adherence to Russell & Burch's [4] Three Rs tenet (replacement, reduction and refinement), a concept that originated from the scientific community as a way to guide researchers in the ethical use of animals in science [5]. Briefly, replacement refers to methods which avoid or replace the use of animals in an area where animals would otherwise have been used; reduction refers to any strategy that will result in fewer animals being used; and refinement refers to the modification of husbandry or experimental procedures to minimize pain and distress (for more complete descriptions see CCAC Three Rs microsite http://threers.ccac.ca/en/alternatives/index.html). The Three Rs is a widely accepted cornerstone of Canadian and international policies on animal-based science [6] and national agencies are beginning to provide more focussed Three Rs resources for researchers. In the United Kingdom (UK) this resulted in the creation of a national Three Rs centre, the National Centre for the Replacement, Refinement and Reduction of Animals in Research (NC3Rs). The NC3Rs mission is “to use the 3Rs to support science, innovation and animal welfare in the biosciences” [7]. Similarly, the CCAC recently launched a Three Rs Program, which is intended to support greater implementation of Three Rs in Canada by providing a focus for Three Rs-related initiatives [8]. However, in order to be effective policymakers require greater understanding of how the Three Rs are viewed by the individuals directly affected by requirements to carry out their research within an ethical framework based on the Three Rs.

Commentary papers and letters to scientific journals have provided some insight into the Three Rs as viewed by individual researchers [9], [10], however these are difficult to generalize from as they are personal perspectives. Another source of insight is from studies of animal ethics committees (AECs), which are composed mainly of animal-based researchers, and several such interview-based studies of Canadian AECs have been conducted [11], [12], [13]. Surveys that examined researchers' views on alternatives and the Three Rs have also been conducted. These have included a survey of UK animal-based researchers to determine the effect of the ethical review process [14]; a survey of European researchers, animal protectionists, ethicists, and regulators views on Three Rs [15]; a survey of UK researchers views on Three Rs [16]; and a survey of laypersons, ‘animal welfarists’ and researchers’ views on animal welfare and alternatives [17].

Of these, only one survey was initiated as part of a policy development process by the UK's NC3Rs [16]. Following on the example of this survey and our own need to generate Canadian benchmark data to measure the effect of the new Three Rs Program, the CCAC initiated the survey reported here. The aim was to understand ‘principal investigators’ and ‘other researchers’ (i.e. graduate students, post-doctoral researchers etc.) views on the Three Rs and also to identify obstacles and opportunities for continued implementation of the Three Rs in Canada.

## Methods

### Ethics statement

This survey was conducted with ethics approval from the Behavioural Research Ethics Board of the University of British Columbia (UBC) (approval number H06-80532). Upon signing into the UBC “YourViews” online survey platform, participants were presented with a consent form and required to accept the terms before they could proceed to the survey. The research team did not have access to participants' names or email addresses and information concerning the participants' home institutions was not collected.

### Survey design

This web-based survey was delivered using the UBC “YourViews” online survey platform created to engage people on ethical issues in science and technology (including animal research, genomics and use of robotics) [18]–[20]. This platform provides a means of collecting survey participants' views with a unique format: “reason-based” questions.

**Figure 1:**
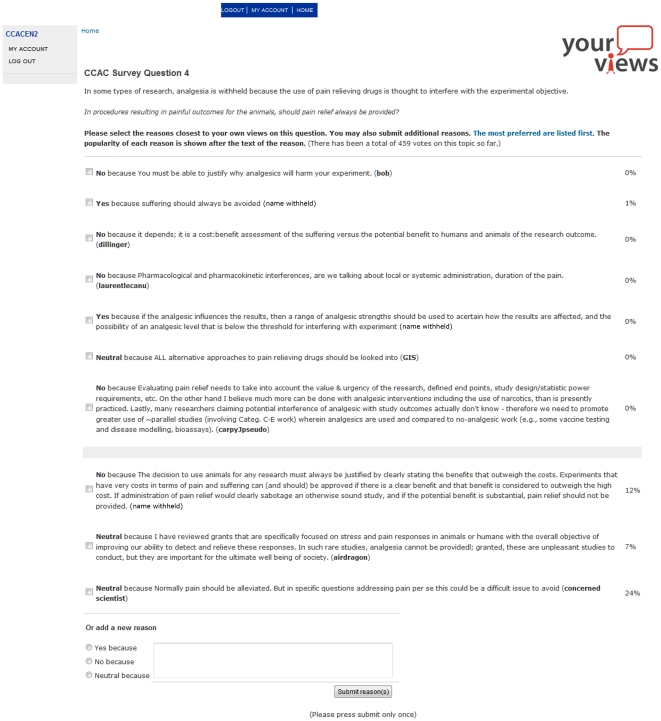
Screenshot of N-reasons interface for survey Q4. Some reasons are omitted; some participant pseudonyms are withheld to maintain participant confidentiality.

The reason-based questions, called “N-reasons”, presented brief hypothetical scenarios or statements followed by a question. Participants were asked to answer the question by choosing (voting) for Yes, No or Neutral. Along with their vote, participants had to provide an explanation (reason) for their vote (text input was required to complete each question and move on to the next). The reasons were displayed on-screen for subsequent participants to read. All but the first participant also had the option of voting for an existing reason that was posted by a previous participant (see screenshot in Figure 1). There was no need to generate conjoint reason responses (i.e. “I agree with reasons 3 and 12”), as participants had the option of voting multiple times within a question for more than one of the displayed reasons (multiple votes were fractionalized, so one participant = one vote).

The features of this design are that it: 1) generates decisions obtained by summing votes; 2) generates answers that explain voting decisions; and 3) allows participants to contribute comments that survey designers did not anticipate (including critiques of the survey questions). Further explanation of the development, experimental validation and testing of the “YourViews” survey platform is described in detail elsewhere [18]–[20].

Our survey consisted of nine main questions: seven N-reasons (described above) and two conventional open-ended questions, as well as demographic questions (for survey questions see Table 1). The open-ended questions required participants to type responses into a text box (i.e. with no voting and these responses were not displayed on-screen to other participants). The survey was prepared in two languages, French and English. Questions were developed in English in consultation with a small group of Canadian animal-based researchers. The questions were then translated to French. Prototype online surveys (one French, one English) were then pilot-tested with a group of approximately 20 researchers in February 2010, and were subsequently revised to incorporate suggested changes.

**Table 1:** Survey questions.

No.	Question
1	An investigator had the option of using tissue from humanely killed rats (purchased for the experiment) or donated human tissue in their experiment. The source of the tissue did not matter scientifically. The investigator decided to use rat tissue because using human tissue required additional administrative steps before the study could proceed. *Is the investigator's decision justified?*
2	An investigator received approval from the animal care committee to use 20 rainbow trout in a cancer research protocol. After a last-minute consultation with a biostatistician, the investigator learned that if the study design was changed, only 15 animals needed to be used to obtain the same result. However, the investigator decided to go ahead with the study as planned and use the 20 trout. *Do you agree with the investigator's decision?*
3	*Would you change your answer [in question 2] if the research animals were beagles rather than trout?*
4	In some types of research, analgesia is withheld because the use of pain relieving drugs is thought to interfere with the experimental objective. *In procedures resulting in painful outcomes for the animals, should pain relief always be provided?*
5	Some investigators try to minimize the total number of animals by re-using animals. *Should animals be re-used in research protocols?*
6	One way to improve animal welfare outcomes for animals used in scientific research is to add pilot studies to the main experiment to determine the impact of these different procedures on the experimental endpoint. *Would you consider conducting an animal welfare-related pilot study prior to, or in parallel with your main research?*
7	Some efforts to implement animal welfare initiatives in research may lead to increased financial costs. For example, the establishment of cell cultures to raise monoclonal antibodies, rather than using mice. *Should financial cost be a factor when making decisions about animal welfare initiatives?*
8	When animal use protocols are prepared, investigators are expected to review ways that they can replace, reduce and refine animal use. *When should assistance with replacement, reduction and refinement be provided to researchers?*
9	*In your research, what is the biggest obstacle to the replacement, reduction and refinement of animal use?*

Upon initially signing into the “YourViews” online survey platform participants were required to enter a username and email address and provide basic demographic information (gender, age category, country of residence and country of origin). They were also required to create a pseudonym and were cautioned that the pseudonym may be displayed on-screen in the survey (for example if they create a new response in a reason-based question). The nine main survey questions followed and additional demographic questions were asked at the end. The first post-survey demographic question asked participants to classify their role in animal-based research by selecting either “I am not listed on animal use protocols”, “Principal Investigator”, or “Other”. Those who selected “Other” were prompted with a text box to describe their exact role. All participants were also asked to indicate the number of years involved in animal-based research; to describe the field of research they work in; and whether they have ever been a member of an AEC.

### Participant recruitment

Recruitment of participants was conducted via third parties. Email invitations with introductory information and an internet link to the survey were sent to the third parties for distribution to animal-based researchers. They included Canadian AECs and university research offices, Canadian granting agencies, and the Canadian Society of Zoologists. A link to the survey was also posted on the homepage of the CCAC website.

Distribution was hampered because some institutions were reluctant to forward the survey due to: concerns that researchers were already over-saturated with survey requests; institutional policy that prevented distribution of surveys; and concerns about the ethical review status of the survey. We asked the third parties to email us with their estimates of how many people the invitation was forwarded to. With this feedback we estimate that approximately 3392 individuals received the invitation emails asking them to participate.

### Data collection

Data was collected in May and June 2010. Various technical problems occurred in the data collection phase of the survey. First, the online platform partially “froze” for approximately a 24 hour period midway though data collection, during which participants were not able to complete the post-survey demographic portion of the survey. Second, the online message to participants that described how the votes-per-reason were tallied and displayed was incorrect. The original message stated that tally display was calculated by an algorithm that weighted both how recently the reason was posted and its popularity. However in practice, the most recently posted reasons were positioned at the top of the screen and the earlier posted reasons were bumped down so that participants would need to scroll down to read them.

Thirdly, for some questions in the English survey, so many individual reasons were submitted that the maximum number of reasons which could be displayed on-screen at once (40) was exceeded. In these cases, the earliest posted reasons were bumped off the screen and replaced by newer postings. No data were lost, however, it meant that participants who completed the survey toward the end of the data collection period would not have had an opportunity to view or vote for those early reasons.

### Analysis

We used the post-survey demographics as a way to filter participants and, as much as possible, ensure that we report the views of our target participants: principal investigators (PIs) and other researchers (ORs). Therefore, responses from participants who indicated they had never been involved in animal research or who identified themselves as never having been listed on an animal use protocol (a requirement in Canada) were excluded from analysis. Similarly, those who did not complete the post-survey demographics (for example because they dropped out by choice or due to the technical problems described above) were also excluded. However, although their votes were excluded from final analysis, if an excluded participant wrote a reason that other participants voted for, then their reason (but not vote) was retained as it was required to analyze the non-excluded participants' selections.

All French responses were translated to English for analysis. Data were analyzed using three different methods. First, for questions 1 to 7 we used the votes to generally characterize views on a topic. The number of Yes, No and Neutral votes by PIs and ORs were summed and percentages calculated. In cases where a participant voted multiple times in one question, their votes were fractionalized to ensure that each participant only cast a total of one full vote per question. Votes from the French and English versions of the survey were combined for this part of the analysis.

**Figure 2:**
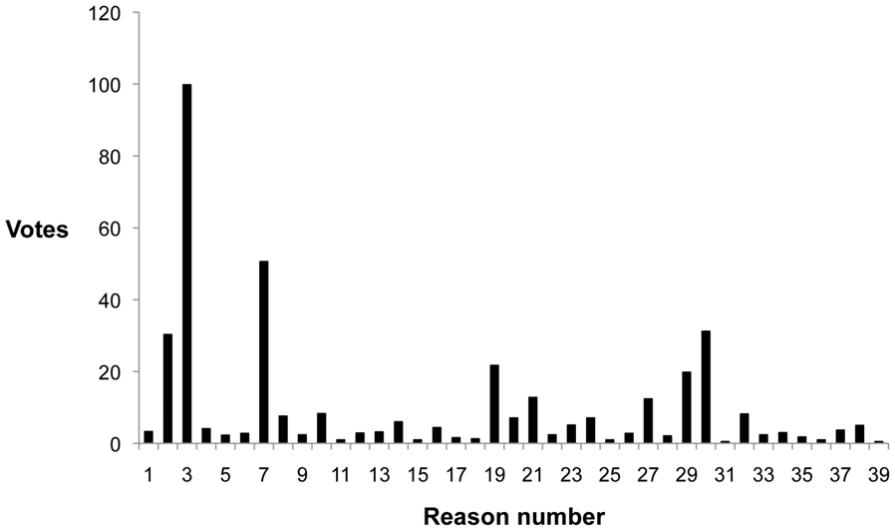
Number of votes per ‘reason’ for survey Q4. Question 4: In procedures resulting in painful outcomes for the animals, should pain relief always be provided?

Second, to expand our understanding of the votes in Q1–7 we then analyzed additional information provided by the reasons.To determine the most “popular” explanations for votes, the reasons were sorted by counting the number of votes per reason. Reasons with the highest proportion of votes were interpreted as representing the most popular reasons associated with each type of vote (i.e. Yes, No or Neutral). For example, in Figure 2 reason numbers 3 and 7 received the highest number of votes and are therefore interpreted as the most popular explanations for voting choices in response to Q4. With this method, reasons from the French and English versions of the survey were analyzed separately (as participants in the English survey would not have been able to view or vote on reasons posted to the French survey and vice versa). In this part of the analysis, votes from ORs and PIs were combined.

As described above, for questions 1, 2 and 5 in the English survey, the number of individual reasons posted exceeded the maximum number of reasons (40) that could be displayed on-screen. To correct for this, in English Q1, Q2 and Q5 we only included reasons posted up to and including the 40^th^ reason in our analysis of reason popularity. This resulted in a smaller sample size for reasons analysis for these questions only. (This correction or smaller sample sizes did not apply to the separate analysis of the absolute votes for Yes, No or Neutral).

The third method of analysis was qualitative analysis of the open-text box responses to Q8 and Q9. This involved reading the comments and developing lists of codes to organize the data. Codes related directly to the question being asked and also arose from the data [21]. Due to the open-ended nature of the questions participants made many comments that were not related directly to the question asked. Applying a qualitative research methodology, these unsolicited responses were coded and analyzed as part of the data. In some cases, frequency counts were also done to determine relative proportions of types of comments (i.e. the number of responses per code was counted). Responses from PIs and ORs were not considered separately in this section of analysis.

### Selection of quotes

To ensure that participants' views can be properly evaluated by readers of this paper, we report participant reasons as written and uncorrected for spelling, grammar etc. (English), or as translated, following standard translation conventions (French). We selected reasons (or quotes from within a reason) to report based on both the popularity of the reason and how well it explained a voting choice. For each N-reason question (Q1–7) we report the most popular reason from the English survey as determined by the numerical proportion of votes. We also include other popular reasons from English and/or French responses without reporting numerical proportions to avoid presenting artificial comparisons between the responses of French language and English language participants. We also reviewed the ‘less popular’ reasons to determine if there were other substantially different explanations for voting Yes, No or Neutral. However, when presenting a reason without data on numerical popularity we use the general terms of many, few, etc. to indicate representativeness. Quotes from Q8 and Q9 were selected on the basis of being the most representative of the codes.

## Results

### Participant demographics

A total of 414 participants were included in this analysis, including 298 (72%) PIs and 116 (28%) ORs (a further 332 participants were excluded for reasons described above). The French version of the survey was completed by 48 participants and 366 participants completed the English version. Calculation of an accurate response rate is not possible as the number of people who received or viewed the invitation to the survey is not known. However, if we use the estimated number of people who received the email invitation (3392) then the response rate estimate is 12.2%. We can also approximate the response rate of PIs using CCAC's estimated number of animal use protocol authors who are PIs for 2008 which was 4600 (CCAC, unpublished data). Therefore, the response rate estimate for PIs is 6.5%. Full demographic information on survey participants is shown in Table 2.

**Table 2:** Demographic information on survey participants: principal investigators (n = 298), other researchers (n = 116) and combined (n = 414).

Demographic category	Number of principal investigators, n = 298	Number of other researchers, n = 116	Combined, n = 414
*Gender*			
Female	92 (30.9%)	76 (65.5%)	168 (40.6%)
Male	201 (67.5%)	39 (33.6%)	240 (58.0%)
Not specified	5 (1.7%)	1 (1.0%)	6 (1.4%)
*Age*			
19–29	3 (1.0%)	40 (34.5%)	43 (10.4%)
30–39	46 (15.4%)	38 (32.8%)	84 (20.3%)
40–49	104 (34.9%)	18 (15.5%)	122 (29.5%)
50–59	96 (32.2%)	18 (15.5%)	114 (27.5%)
60-above	47 (15.8%)	1 (0.9%)	48 (11.6%)
Not specified	2 (0.7%)	1 (0.9%)	3 (0.7%)
*Education*			
College/university	15 (5.0%)	35 (30.2%)	50 (12%)
Masters	11 (3.7%)	35 (30.2%)	46 (11%)
Doctorate	268 (89.9%)	46 (39.7%)	314 (76%)
Other	4 (1.3%)	0	4 (1.0%)
*Years of experience in animal-based research*			
<10 years	15 (5.0%)	38 (32.8%)	53 (12.8%)
10–20 years	142 (47.7%)	61 (52.6%)	202 (48.8%)
>20 years	141 (47.3%)	17 (14.7%)	158 (38.2%)
*AEC Membership*			
Never a member	164 (55.0%)	97 (83.6%)	261 (63.0%)
Past or current member	134 (45.0%)	19 (16.4%)	153 (37.0%)
*Research area* *			
Agricultural	9 (3.0%)	2 (1.7%)	11 (2.7%)
Animal welfare and health	23 (7.7%)	1 (0.9%)	24 (5.8%)
Behaviour	25 (8.4%)	4 (3.4%)	29 (7.0%)
Biology	40 (13.4%)	18 (15.5%)	58 (14.0%)
Ecology	24 (8.1%)	5 (4.3%)	29 (7.0%)
Genetics	13 (4.4%)	2 (1.7%)	15 (3.6%)
Human diseases	64 (21.4%)	40 (34.5%)	104 (25.1%)
Immunology	12 (4.0%)	10 (8.6%)	22 (5.3%)
Neuroscience	60 (20.1%)	24 (20.7%)	84 (20.3%)
Pharmacology, drug & vaccine	16 (5.4%)	9 (7.8%)	25 (6.0%)
Physiology	29 (9.7%)	11 (9.5%)	40 (9.7%)
Toxicology	9 (3.0%)	2 (1.7%)	11 (2.7%)
Wildlife	23 (7.7%)	6 (5.2%)	29 (7.0%)
Other	28 (9.4%)	19 (16.4%)	47 (11.4%)

*participant could respond with more than one research area.

Almost all participants were residents of Canada except for 7 who listed another country as their residence or did not answer. Most participants listed Canada as their country of origin (Canada 65%; Europe 17%; US 8%; other 10%). Many participants were involved in more than one research area, so that the sum of researchers in all areas exceeds the number of participants: 25.1% participate in research on human disease, 20.3% in neuroscience and 14.0% in biology.

In our sample of PIs: 30.9% were female and 67.5% male; age varied from 19–29 (1%), 30–39 (15.4%), 40–49 (34.9%), 50–59 (32.2%) to greater than 60 (15.8%); education varied from college/university (5.0%), Master's degree (3.7%) and Ph.D. degree (89.9%); years of experience in animal-based research varied from less than 10 (5.0%), 10–20 (47.7%) to greater than 20 (47.3%); 55.0% had never been a member of an AEC.

In our sample of ORs: 65.5% were female and 33.6% male; age varied from 19–29 (34.5%), 30–39 (32.8%), 40–49 (15.5%), 50–59 (15.5%) to greater than 60 (0.9%); education varied from college/university (30.2%), Master's degree (30.2%) and Ph.D. degree (39.7%); years of experience in animal-based research varied from less than 10 (32.8%), 10–20 (52.6%) to greater than 20 (14.7%); 83.6% had never been a member of an AEC. The composition of ORs was 23.3% graduate students, 5.2% post-doctoral fellows, 9.5% research associates, 25.9% research technicians and assistants and 35.3% other roles.

### Votes and reasons

As explained above, we report reasons (or quotes from within a reason) based on both the popularity of the reason and how well it explained a voting choice. We identify quotes with the reason identification number (rid) in parenthesis after the quote. For some quotes we report the percentage of votes and question sample size, also in parenthesis after the quote. All quotes are presented in italics.

**Figure 3:**
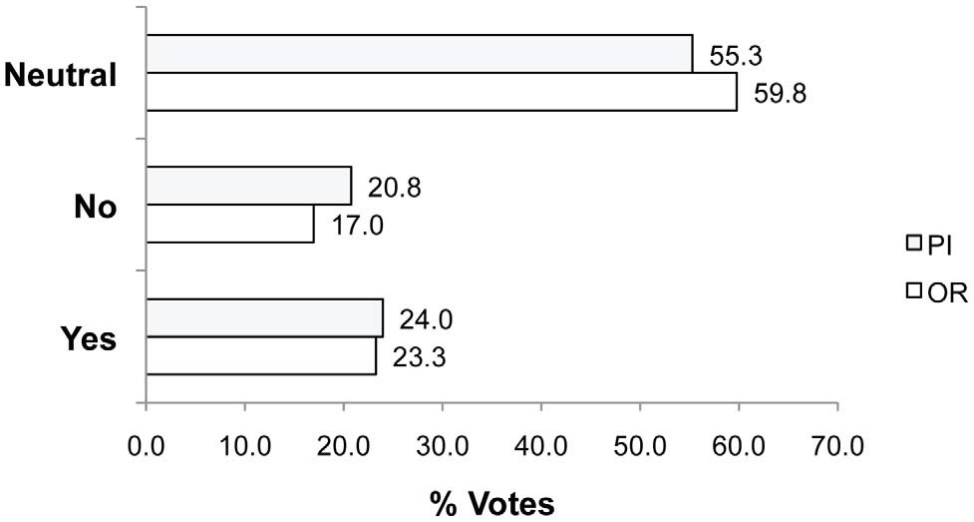
Percent of Yes, No and Neutral votes in response to Q1. Question 1: Is the investigator justified in using animal tissue instead of pursuing the possibility of human tissue use?

In Q1 we asked participants whether they agreed if a hypothetical researcher was justified in using animal tissue instead of pursuing the possibility of using human tissue. Most participants voted Neutral (Figure 3, 55.3% PIs; 59.8% ORs), while the remainder were split between Yes and No. Participants chose to vote Neutral because not enough information about the hypothetical experimental objectives was provided. For example, **Neutral because**: *“It depends on the hypothesis to be tested. If the hypothesis is pertinent to humans specifically, the investigator should have used human tissue. On the other hand, if the hypothesis is not specific to humans, then the rat tissue is perfectly acceptable and justified because the work could proceed more quickly”* (rid 2217; 23.6% of Q1 votes; n = 255).

Other participants who voted Neutral also commented about the lack of information about factors such as extra time and money costs. Others found the question “*leading*” or “*ill-conceived*”. Twenty-four percent of PIs and 23.3% of ORs agreed that the use of the animal tissue was justified (voting Yes). This group referred to the limited availability of human tissue for example, **Yes because**: *“… if the two types of tissue are equivalent for the experiment, it could be preferable to keep the human tissues for experiments where they cannot be replaced by animal tissues”* (rid 2256; translated from French). In contrast, those voting No (20.8% PIs; 17.0% ORs) explained, **No because**: *“It is not permissible to sacrifice an animal merely for the sake of convenience”* (rid 2242).

**Figure 4:**
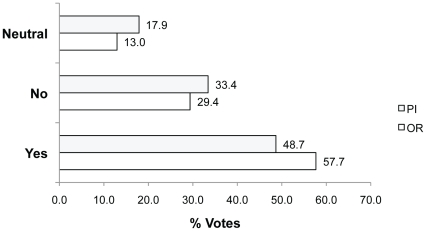
Percent of Yes, No and Neutral votes in response to Q2. Question 2: Do you agree with the investigator's decision to use 20 trout (in an approved protocol) instead of pursuing the possibility of using just 15?

In Q2, we asked whether participants agreed with a hypothetical researcher's decision to still use 20 rainbow trout after learning that with a study design change, only 15 animals could be used to obtain the same result. Most participants agreed (voted Yes) with the hypothetical researcher (48.7% PIs; 57.7% ORs) or were Neutral (17.9% PIs; 13.0% ORs) (Figure 4). Their reasons suggest it is because they prefer to use enough animals to ensure quality data is obtained rather than using the absolute minimum and potentially wasting these if something goes wrong. For example, **Yes because**:*“a biostatistician is only going to be able to give you an ESTIMATE of statistical power based on certain assumptions of the statistical test, variability among animals, and the desired “effect size” to be detected, which is often arbitrary. Using a few more animals is erring on the side of caution”* (rid 2255; 18.4% of Q2 votes; n = 333). Similarly another reason explained, **Yes because**: *“This implies that 5 more trouts and a sufficient statistical power remain probable. It is best to sacrifice 20 trouts than only 15 and be almost certain not to sacrifice 15 for nothing”* (rid 2240).

Those voting Neutral cited both the lack of information in the question and frustration with the criticism of a scientist carrying out a protocol that had already been approved by an AEC. For example, the following reason explained **Neutral because**: *“…One would have to assume that the original plan for 20 was based on advice from a biostatistician and/or a solid rationale and this would be necessary in order to get approval in the first place. Therefore, at best, this scenario means that one of the two biostatisticians is wrong. Passing judgement on the investigators choice is not really justified there would be some consideration of circumstances and evaluation prior to reaching a decsision [sic]. We are not given the circumstances.”* (rid 2275). Those disagreeing (voting No, 33.4% PIs; 29.4% ORs) with the use of 20 rather than 15 animals mostly commented that if fewer animals could be used then that is what should be done. For example, **No because**: *“This is completely contrary to the 3Rs. Only the minimum number of animals required should be used”* (rid 2243).

**Figure 5:**
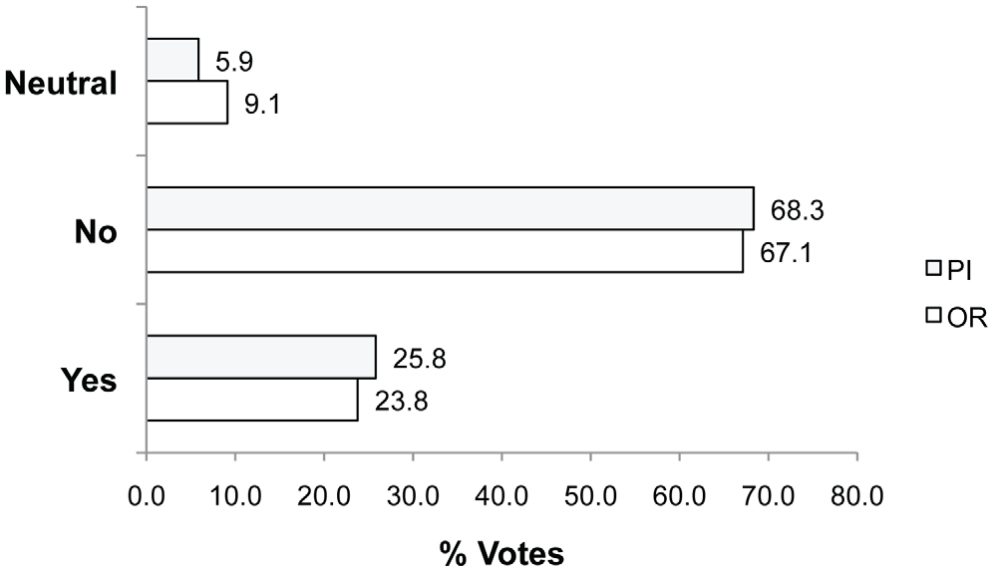
Percent of Yes, No and Neutral votes in response to Q3. Question 3: If the experimental species in question 2 was beagles instead of trout would you change your response?

The third survey question (Q3) asked whether participants would change their vote in Q2 (above) if the experimental species changed from trout to beagle. Participants who voted Yes to Q3 were indicating that species would make a difference in how they would respond to Q2 (which asked whether an investigator was justified in using 20 instead of 15 animals). About one quarter PIs (25.8%) and ORs (23.8%) voted Yes they would change their vote (Figure 5). The reasons for why species would change a participants vote were varied but included practical considerations, (such as cost of animal care), and ethical issues. Less mentioned reasons related to personal choices and concerns about the value of research that could switch between such different species. For example, one popular reason explained **Yes because**: “*Beagles are much more expensive than trout - I am sure 1 beagle costs 10× the cost of 20 fish and the daily housing costs would be also far most significant. Also, the chances of loosing [sic] 5 beagles due to problems with housing are extremely slim because the individual animals are monitored MUCH more closely than are fish*” (rid 2219). Another said, **Yes because**: “*Although the 3Rs suggest that we should use the least number of animals possible, I feel more and more obliged to use as least as possible when it comes to higher order animals*” (rid 2381).

Most participants voted No (i.e. they would not change their vote cast in Q2, whether Yes, No or Neutral) (68.3% PIs; 67.1% ORs).The most popular reasons were **No because**: “*the same logic applies*” (rid 2268; 14.6%; n = 366) and **No because**: “*the species does not affect my decision*” (2257, translated from French). However, these reasons do not give any clues about how participants initially voted in Q2. As reported above, most participants were in favour of using more animals as a precaution to ensure that experimental data does not go to waste, and some responses to Q3 suggest that view would not change based on species. For example, **No because**: “*in the context of research, an animal is an animal whether it is a trout or a dog. The 3 R's principles applies to all vertebrates …as researchers we must assure that our use of animals in research results in valid outcomes and not assurance of using the absolute minimum number of animals in our experiments. Even with power analyses, one can never know with very high confidence the exact minimal sample size; we need to err on the side of caution which often means slightly larger sample sizes*” (rid 2326) and, **No because:** *“…we cannot decide these matters on the ‘cuteness’ factor of the animals involved”* (rid 2223).

**Figure 6:**
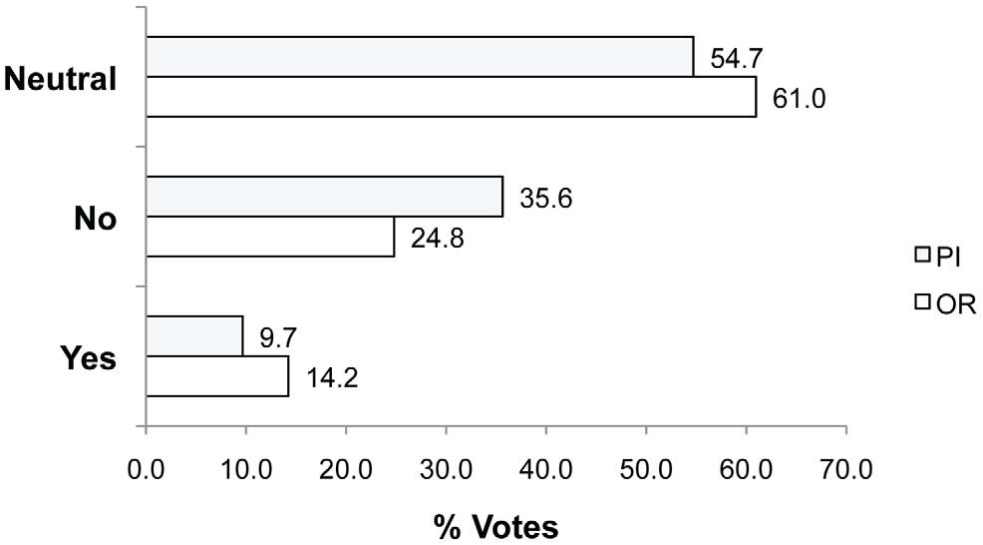
Percent of Yes, No and Neutral votes in response to Q4. Question 4: In procedures resulting in painful outcomes for the animals, should pain relief always be provided?

In Q4, participants were asked whether “pain relief should always be provided to animals during painful procedures”. Only a small proportion voted Yes (9.7% PIs; 14.2% ORs), mainly because they felt that there was no justification not too and/or because they believed pain could be detrimental to the experimental outcomes (Figure 6). For example, **Yes because:** *“Pain relief should always be provided. There is no justification for making the animals suffer since stress caused by pain will also interfere with experiment”* (rid 2379).

The majority of participants felt that there are some situations where use of pain relief may interfere with experimental outcomes (voting Neutral, 54.7% PIs; 61.0% ORs; or No 35.6%PIs; 24.8% ORs). However, they also acknowledged that justification should be strong for experiments that do not provide pain relief and pain should be minimized as much as possible.

For example, **Neutral because**: “*Normally pain should be alleviated. But in specific questions addressing pain per se this could be a difficult issue to avoid*” (rid 2224, 26.0%, n = 366) and **Neutral because:** *“If testing the effects of pain, for example, analgesia cannot be given before. However, these studies must be elaborated in detail and closely controlled and analgesia must be given as soon as possible”* (rid 2234).

Similarly, this participant voting No explained **No because**: “*The decision to use animals for any research must always be justified by clearly stating the benefits that outweigh the costs. Experiments that have very costs [sic] in terms of pain and suffering can (and should) be approved if there is a clear benefit and that benefit is considered to outweigh the high cost. If administration of pain relief would clearly sabotage an otherwise sound study, and if the potential benefit is substantial, pain relief should not be provided*” (rid 2263).

**Figure 7:**
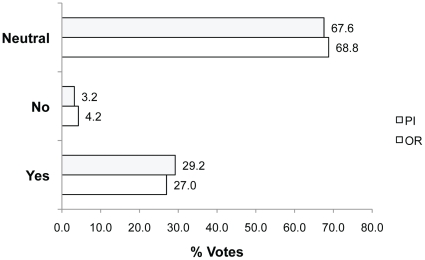
Percent of Yes, No and Neutral votes in response to Q5. Question 5: Should animals be re-used in research protocols?

When asked whether animals should be re-used in research protocols (Q5), most participants voted Neutral (67.6% PIs; 68.8% ORs) and their reasons suggest concern about how re-use might compromise experimental objectives (Figure 7). For example, **Neutral because**: “*Sometimes re-using animals works, but sometimes the entire experiment could be ruined by re-using the animals. In the latter case, it would be most effeicient [sic] not to waste the investigators time, monetory [sic] resources and the animal's lives by improperly re-using the experimental animals*” (rid 2225, 25.8% of votes, n = 333).

Those who indicated support for re-use (i.e. by voting Yes, 29.2% PIs; 27.0% ORs) mostly qualified their vote with criteria for instances when re-use is appropriate. For example, **Yes because:** “*Absolutely. Providing: due regard is given to the invasiveness of the studies. i.e. re-using animals following surgical studies may not be appropriate [and] re-use is scientifically acceptable [and] an appropriate washout/rest period is provided between studies*” (rid 2248). Similarly, **Yes because**: “*some experiments, not invasive or less invasive, make re-using animals possible. This would not be acceptable for invasive experiments*.” (rid 2652, translated from French). Few participants voted No (3.2% PIs; 4.2% ORs) citing both scientific and animal-welfare concerns. For example, **No because**: “*not only are you adding another layer of complexity to your analysis, you are submitting the animal to anoter [sic] set of stressful situations*.” (rid 2332).

**Figure 8:**
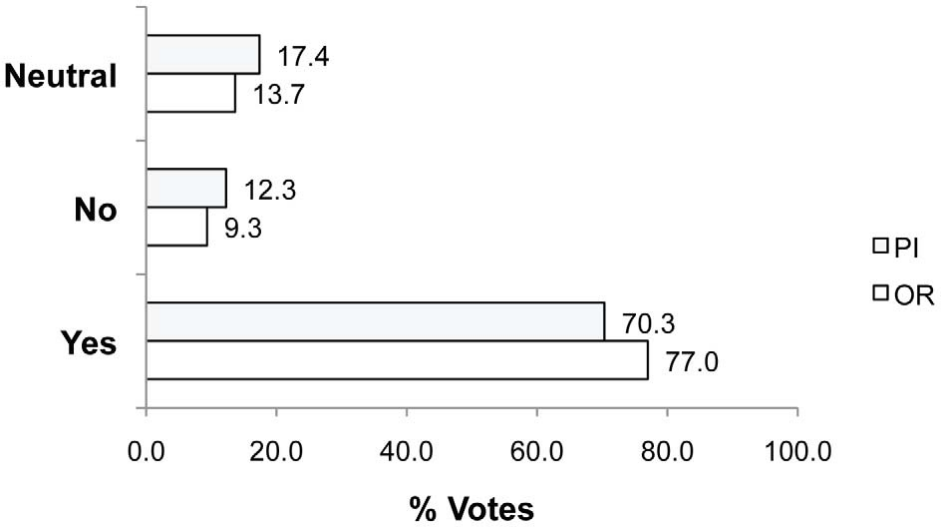
Percent of Yes, No and Neutral votes in response to Q6. Question 6: Would you consider conducting an animal welfare-related pilot study prior to, or in parallel with your main research?

We also asked whether researchers were open to conducting animal welfare-related pilot studies (Q6). There appears to be support for this as the majority (70.3% PIs; 77.0% ORs) voted Yes they would consider conducting an animal welfare-related pilot study prior to or in parallel with their main research (Figure 8). Generally, participants felt that this approach had advantages for both scientific outcomes and animal welfare. For example, **Yes because**: “*Adding a pilot study to determine an appropriate endpoint for a new procedure is ethically very sound. However, the pilot project should be conducted PRIOR to commencing work on the main project. In the end, this pilot project will help not only by maximizing animal welfare, but might also be used for power calculations so the overall number of subjects could be minimized*” (rid 2306; 26.0% of votes; n = 366). Another explained, **Yes because:** “*This allows [us] to establish the real needs of the experiment*” (rid 2236, translated from French).

Neutral voters (17.4% PIs; 13.7% ORs) advocated a more cautious approach in their responses. For example, **Neutral because:** “*pilot studies are not always appropriate. If a pilot can be done so that the results can be merged with the overall study results, then it is justifiable. But if the pilot results serve no purpose other than measuring animal welfare outcomes, it may be a case of harming more animals than necessary. So in my opinion, pilot studies must never be default requirements for research approval. They must be judged individually for appropriateness*” (rid 2374).

The smaller proportion of participants who were opposed to animal-welfare pilot studies in their own research (voting No) (12.3% PIs; 9.3% ORs) were concerned about the appropriateness of diverting money from their research grants. For example, **No because:** *“As long as the experiment is according to ethical norms I will not do this as a side project. It involves research money, time and labor that are not feasible within the mandate of my operating grant”* (rid 2269) and **No because:** “*If the subsidized research theme is not animal welfare, I do not see why doing [sic] pilot studies on that topic*” (rid 2334, translated from French).There was also concern about the extra time and resource burden on the researcher, and the fact that in some research sample sizes are so small a pilot study could not be done.

**Figure 9:**
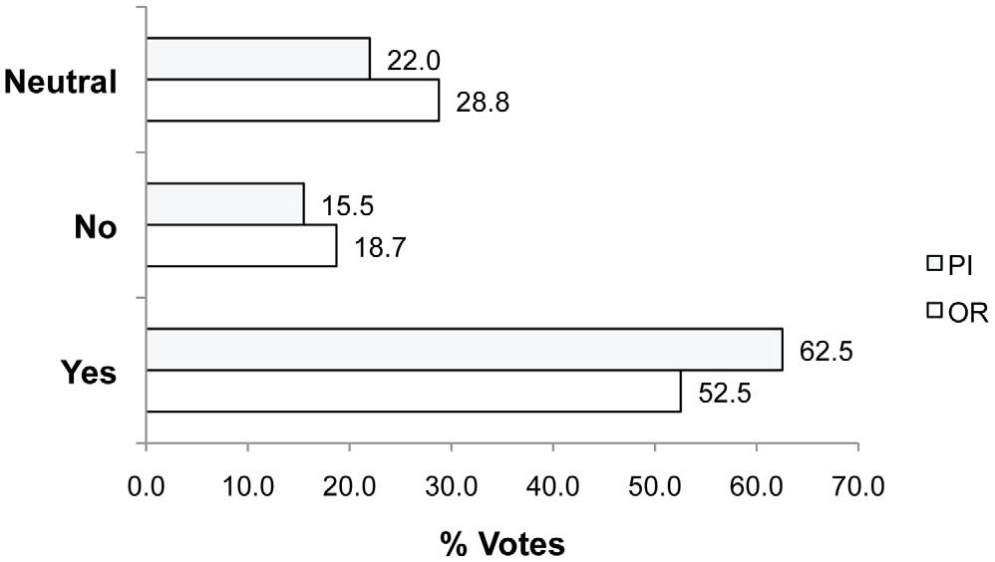
Percent of Yes, No and Neutral votes in response to Q7. Question 7: Should financial cost be a factor when making decisions about animal welfare initiatives?

We also asked if financial cost should be a factor in decisions about animal welfare initiatives (Q7). Most voted Yes (62.5% PIs; 52.5% ORs), indicating that the financial cost of an animal welfare initiative is an important consideration for Canadian researchers (Figure 9). For example, **Yes because**: *“Finances should be ONE of MANY factors when making decisions about animal welfare initiatives”* (rid 2229, 39.9% of votes, n = 366). Those voting Neutral (22.0% PIs; 28.8% ORs) said for example, **Neutral because**: “*Within reason, cost should not be a factor… BUT, I doubt that anyone can walk through their vivarium and see something that could be done to improve animal welfare if only enough money was available*” (rid 2252). In contrast, those voting No (15.5%PIs; 18.7%ORs) explained their opposition by saying for example, **No because:** *“cost and welfare are two completely separate issues, and it is a deep moral quagmire to deal with them in the same discussion. If an animal-free alternative of similar usefulness is available, it MUST be used, or the research should not proceed”* (rid 2375).

### Open ended responses

The last two survey questions required participants to type responses into text-boxes. As described above, their responses were analyzed using qualitative methodology and responses from PIs and ORs were not analyzed separately. Quotes are identified by user identification number (uid) and whether from a PI or OR in parenthesis.

When Canadian researchers submit animal use protocols for ethical review they are expected to have reviewed the ways that they could implement the Three Rs. Therefore, we asked participants to comment on when “assistance” with replacement, reduction and refinement should be provided (Q8). Responses indicated that there was some confusion over the meaning of this question, however 306 participants responded by describing a stage (or multiple stages) in the research process where Three Rs-related assistance might be useful and/or acceptable. These stages and the number of times each was mentioned are listed in Table 3. Proportions suggest that more participants would find Three Rs-related assistance acceptable during preparation of animal use protocols (25%) and during or after the animal use protocol review process (29%). A small proportion (7%) of participants felt that assistance should not be provided.

**Table 3:** Stage in research process where Three Rs-related assistance might be useful and/or acceptable, n = 306.

Stage in research process	Times cited
Training on animal use	24 (8%)
Research planning (grant applications review; peer review)	28 (9%)
During preparation of animal use protocols	76 (25%)
During/after the animal use protocol review process	90 (29%)
When a problem is identified in the protocol	33 (11%)
When PIs ask for assistance	47 (15%)
On-going assistance should be provided	47 (15%)
Other	33 (11%)
Assistance should not be provided	20 (7%)

In their responses to Q8, almost one quarter of survey participants (86) commented on the type of Three Rs assistance that would be acceptable. This included a resource or resources (i.e. guideline, database, website) that outline specific Three Rs alternatives. Many emphasized that any assistance should be “*constructive*”, “*neutral*” and not mandatory. A few suggested that assistance should only be offered if a particular problem was identified with the research or the researcher. About 15% of participants (62) also commented about who should be providing Three Rs assistance. Of these responses, about half emphasized that Three Rs assistance must be delivered by someone with scientific expertise in the specific research area they are advising on. For example, “…*replacement, reduction and refinement advice should be provided to researchers only by qualified personnel, otherwise it won't be taken seriously*.” (uid 4906, OR) and *“Familiarity with the protocols and literature in an area is seemingly crucial for dictating changes in protocol”* (uid 4655, PI).

However, opinion diverged over which individuals or groups possessed the correct level of scientific expertise. The following series of quotes illustrate opposing opinions about the expertise of AECs: *“Animal care committees are probably NOT the most informed about alternatives to animal use. Internal peer review panels are perhaps better sources of ideas to researchers about replacement, reduction and refinement”* (uid 4829, OR); and *“… If the investigator does not offer adequate justification, the committee must informed [sic] the investigator of it and offer him the assistance of a professional. I do not believe in the competence of the committees to make acceptable suggestions.”* (uid 5341, PI, translated from French).

In contrast, “*I feel that investigators are left to grope around for answers on their own when preparing protocol applications. The animal committee has the experience and wide resources….it would be helpful if they made suggestions that can be incorporated*” (uid 4980, OR); and, *“…The institutional ACC should also try to offer workshops (e.g. during professional development days) to educate the University community about issues related to the 3 Rs. Once the researcher begins writing the protocol, the Research Officce [sic] and the ACC (specifically, the ACC Chair, ACC coordinator, and the ACC veterinarian) should be available to answer additional questions as appropriate”* (uid 4676, PI).

Some participants felt that only PIs have the most suitable expertise explaining, for example, that assistance could come from *“Discussion with the veterinarian, while understanding that the investigator is often in a better position to determine if a model can be replaced or not in a type of research experiment”* (uid 5421, PI, translated from French) and “*the researcher is the expert in that area of work and therefore is best postioned [sic]to provide a response to the question related to the 3R's*” (uid 5113, PI).

We also asked participants to describe the biggest obstacle to Three Rs in their own research (Q9). About half of survey participants (222) responded by identifying obstacles to replacement. Participants said they could not use a replacement alternative citing the following reasons most often: because animals are the subject of the research (i.e. studying wild animals, animal behavior, production animals etc.); because the “*whole animal*” must be used in their research and because there are no replacements available. A smaller proportion of participants also described the dependence of the research on a particular species or long established model, and the difficulty accessing alternatives (such as human tissues) as obstacles to replacement.

About one quarter of participants (110) identified obstacles to reducing animal numbers. Most described the variability of biological systems and requirements for statistical significance of experimental results specific obstacles to reduction. Many also expressed concern that efforts to reduce may compromise their research. A small proportion (about 3%) indicated that refinement was an obstacle. Fewer than 10% of participants reported lack of money as their biggest obstacle to implementing Three Rs in their research.

Emerging from Q9 was the assertion from many participants that they are implementing the Three Rs as much as they can, For example, one researcher wrote: “*I am already working with cells obtained from animals, which is due to reduction. We are using far less number of animals than if we were to do this study in vivo. Replacement is not possible as we need primary cells for our study and it cannot be replaced with cell lines. The procedure is already refined with the use of a proper anesthetic. So the 3R's are already implemented in my research*” (uid 4950, OR). Many participants commented that they are reducing animal numbers to the minimum possible. For example, “*… I have lowered the amount of rats I use as much as possible to still maintain scientific integrity and so I can still find significance if it is there*” (uid 4671, OR); and “*I have always worked on reducing the numbers of fish and have changed my protocol (often) to reduce the numbers of animals that I sacrifice for tissue analysis*” (uid 4653; PI).

A few participants indicated that they could possibly do more in the areas of refinement and reduction. However, others specifically stated that their protocols had reached a stage where further Three Rs changes are no longer possible. For example, one PI commented “*Our protocols are currently refined enough and there is a limit to refinement and reduction*” (uid 5514, PI, translated from French). Another wrote, “*3Rs have been refined in my work by dozens of labs over 80 years and I have contributed my own efforts. So the biggest obstacle now is making them better than they are already*” (uid 4836, PI) and “*As a researcher with large animals, cost is a major factor in conducting research, the incentive to replace, reduce and refine is alreay there. To go any further down that path generally results in poor data, weak conclusions and an inability to publish*.” (uid 5306, PI).

## Discussion

### Representativeness of participants to Canadian animal-based researchers

Our aim was to understand the views of Canadian animal-based ‘principal investigators’ and ‘other researchers’ (i.e. graduate students, post-doctoral researchers etc) however, there is no data source that specifically describes the demographics of these groups. Therefore, we have used other measures to assess the representativeness of our sample. Potential participants could have been researchers from any CCAC constituent group: universities and colleges, industry, or government. However, since there are a greater number of university constituents and almost all of the participants were residents of Canada, we have compared some of our demographic data with Canadian government data on the occupation of University Professors (category label E111).

The gender of our sample of PIs (30.9% female, 67.5% male) corresponds to Statistics Canada data for 2006 that reported 33.1% of university professors in Canada were female [22]. (In contrast, 65.5% of ORs were female and 33.6% were male but we have no way of determining whether our sample is representative of this group). The majority of PIs (89.9%) had PhDs which is usual for university researchers. The remaining PIs may have been from industry, government or college institutions or from a medical or veterinary school where a PhD may not be a pre-requisite for identification as a PI. Not surprisingly, just 39.7% of ORs had PhDs and this was expected as they are trainees. The ages and years of experience in animal-based research were greater for PIs than for ORs. Again, this corresponds with their respective roles and responsibilities in animal-based research. The PIs responding to this survey appear to be slightly older than the national university professor population. For example, approximately 67% of PIs in this survey were in the range of 40–59 years compared to 56% of university professors [22].

We were also interested in the proportion of survey participants who were current or past members of AECs. The majority of participants were not past or present members of AECs (63.0%) and therefore we can be somewhat confident that survey results do not just reflect the views of AEC members, but also those who have never been involved in formal ethical review of animal use protocols. Generally, the demographic data on gender, education, age, country of residence and years of experience in animal-based research is reasonably consistent with our target participants.

### Views on Three Rs

With the initial questions in our survey (Q1–Q3) we intended to illustrate situations where Three Rs needs may conflict with the needs of an animal-based experiment. The hypothetical nature of these questions was not successful with our participants as shown by the high proportion of Neutral votes and accompanying reasons that stated not enough information was provided in the question. The design and content of these questions also received much criticism from participants. However, this was not entirely surprising and may be viewed as both a feature of asking well-informed participants about a topic they specialize in, and of acquiring qualitative information on people's views [23].

We asked about views on replacement in two ways. Q1 was intended to illustrate an example of a conflict between an experimental protocol and replacement. In Q9 we asked participants to identify obstacles to implementing the Three Rs in their research. Responses suggest that researchers hold slightly different views about what qualifies as a suitable replacement alternative when compared to regulators. For example, CCAC policy stipulates that if the scientific objectives of the study can be achieved by using available non-animal models or animals of low sentience then the researcher must consider using these and provide justification for its rejection [24]. In contrast, participants preferred to reserve the potential alternative (in our scenario, human tissue) only for experiments where animal tissue could not be used. However, Q1 did not articulate the details associated with substitution of human tissue (i.e. possible time delays, money, ethics review for using human tissue, possible data ownership issues, biohazard risks etc.). A more detailed scenario may have elicited different views.

Participants also told us that replacement is simply not an option for certain types of animal use, such as when animals are the subject of the research (i.e. studying wild animals, animal behavior etc.); when researchers need to use the “whole animal” and, more generically, when no replacements are available. These findings agree with previous studies. For example, a 2001 UK study found that 84% of surveyed researchers disagreed with the statement “replacement alternatives are better than animal experiments for scientific research” [14] and 50% of those surveyed did not believe that information of equal value to animal experiments can be obtained from Three Rs alternatives. Similarly, researchers who completed a 2008 UK survey also expressed skepticism about the goal of replacement: 77% responded that nothing would enable them to continue their research without using animals as they “need to look at whole animal systems”, and 73% agreed with the statement “Complete replacement of the use of animals in research and testing will never be achieved” [16]. A fourth survey found that researchers agreed significantly less than laypersons and ‘animal welfarists’ in the existence of alternatives to animal use in (medical) research [17]. When UK researchers were asked to identify the main obstacle to implementing Three Rs, the reason given the most was “Lack of appropriate scientific or technological innovation” (33% of responses) [16]. These studies and results from our survey indicate that researchers currently do not view the Three Rs goal of replacement as achievable.

We also asked about reduction, first in Q2 which illustrated a conflict between an experimental protocol and reduction, and again in Q9. Responses suggested that researchers prefer to use enough animals to ensure quality data is obtained rather than using the minimum and potentially waste those animals if a problem occurs during the study (i.e. err on the side of too many rather than too few). Obstacles to reduction included the variability of biological systems and requirements for statistical significance of experimental results. Many participants said that they already reduce as much as possible and had concerns that further reduction may compromise the quality of their research. This finding highlights a potential problem if the reduction efforts of researchers, AECs and policymakers are focused mostly on individual experimental protocols, and less at the level of research programs. Emphasis on a different aspect of reduction may have more support from researchers. For example, UK researchers identified data sharing or collaboration between research groups (77%) and companies (60%) as factors which would allow fewer animals to be used [16]. However, in contrast to these views a recent analysis of experimental design in published animal studies identified many areas for improvement [25], so continued scrutiny of reduction at the experimental level is still valid.

Another theme emerged from responses to Q2: frustration with the ‘second guessing’ of an approved protocol. This frustration occurs when previously approved protocols are up for renewal and reassessment by AECs who then request changes be made (CCAC, personal communication). The Three Rs concept pre-supposes that continuous Three-Rs related improvements can, and should, be made. For example, refinement is viewed as an iterative process [26], and Canadian guidelines require that every protocol be reviewed annually and take into consideration changes in developments in replacement, reduction, and refinement of experimental animal use [24]. However, this appears in conflict with researchers' views about “limits” to Three Rs changes, especially in situations where established protocols are repeated or changes cannot be made, as results may not be comparable with established literature.

Refinement was rarely identified by participants as an obstacle in responses to Q9. However, when we asked about the use of pain-relieving drugs (a refinement) in Q4, most participants felt that there are situations where research animals should not receive pain relieving drugs because it may compromise the experiment. This is comparable to results from CCAC's survey of analgesia-withholding that found researchers justify withholding analgesia, mainly because analgesia has been *proven* conclusively to interfere with experimental results, or *may* interfere with experimental results [27]. Together, these findings identify that research to clarify whether analgesia interferes in specific protocols as well as the development of alternative method of pain relief should be a priority Three Rs issue for both researchers and policymakers.

We were curious to know about researchers' views about the re-use of animals, a practice that can contribute to reduction of total animal numbers but has the potential to increase harms to individual animals (and therefore be in conflict with refinement). Most participants were ambivalent about re-use, and expressed concern that the practice could compromise experimental outcomes. (Fewer participants commented on the potential harms to animals as reason not to re-use). Those who agreed in principle with re-use said their support depended on how invasive the experiments were. Re-use is typically considered acceptable when use is minimally invasive. Some examples include, re-using animals used as controls in a previous study, re-using animals that have been trained to co-operate in routine laboratory procedures (may be less stressful than training new animals) and re-using animals in longitudinal studies [28].

There was strong support from participants for the Three Rs strategy of conducting animal welfare-related pilot studies. Responses to Q6 indicate that researchers viewed pilot studies as useful for both animal welfare and experimental design. However, some cautioned that pilot studies done only to assess animal welfare outcomes may “harm more animals than necessary” and this highlights a potential conflict between reduction and refinement. Not surprisingly, financial costs were viewed as a factor influencing animal welfare initiatives, but responses indicate that researchers do not view this as the only, or dominant factor in animal welfare decisions. Interestingly, many participants who said they would not consider doing animal welfare-related pilot studies explained that this was because they felt it would be inappropriate use of their research funding.

A European survey of how researchers search for Three Rs alternatives found that these researchers would prefer to receive assistance when searching for Three Rs alternatives [29]. Similarly, most participants in this survey were not opposed to being offered assistance to implement the Three Rs, so long as the input is provided in a collegial manner, and from individuals who are experts. Most indicated the timing of assistance should coincide with the preparation of animal use protocols and/or the animal use protocol review process. This view of when Three Rs assistance should be offered differs from the conclusions of Gauthier et al. [30] who suggest it is more relevant to obtain Three Rs input at an earlier stage in the research process, such as grant writing and program of work planning. In addition, 95% of UK researchers reported that they consider the Three Rs when designing and carrying out experiments, while just 60% consider the Three Rs when preparing for the ethical review process [16].

In this study we aimed to understand the views of ORs as many will become PIs in the future. Judging by the patterns of votes in Q1 to Q7, there appear to be no issues where ORs have obviously different views from the PIs in this survey. A possible exception is Q7 where slightly more PIs agreed that financial costs should be a factor in decisions about animal welfare initiatives. However this is not a statistically confirmed difference nor is it surprising as PIs, rather than ORs are typically responsible for research budgets. Overall, views of ORs are similar to PIs. This informs policymakers that despite being more likely to have received training in the implementation of the Three Rs, younger researchers cannot be presumed to have greater acceptance of Three Rs goals when compared to older PIs (who have had to adapt to changes in policy requiring greater implementation of the Three Rs through their careers).

### Conclusion

This survey has provided Canada's peer-based system of policy development with empirically-based insight into researchers' views on the Three Rs, and has identified opportunities for continued implementation of the Three Rs in Canada. Replacement is clearly difficult to implement, therefore occurring less than reduction and refinement. It may be useful for animal-use policymakers to consider what steps are needed to make replacement a more feasible goal. In this context, initiatives that offer researchers greater practical and logistical support with implementation of the Three Rs may be useful. In particular, the strategy of conducting animal welfare-related pilot studies to develop Three Rs knowledge is accepted by researchers. Encouragement and financial support for these initiatives may result in valuable contributions to Three Rs knowledge and improve welfare for animals used in science.
